# The Synergistic Toxicity of Pesticide Mixtures: Implications for Risk Assessment and the Conservation of Endangered Pacific Salmon

**DOI:** 10.1289/ehp.0800096

**Published:** 2008-11-14

**Authors:** Cathy A. Laetz, David H. Baldwin, Tracy K. Collier, Vincent Hebert, John D. Stark, Nathaniel L. Scholz

**Affiliations:** 1 NOAA (National Oceanic and Atmospheric Administration) Fisheries, Northwest Fisheries Science Center, Seattle, Washington, USA; 2 Food and Environmental Quality Laboratory, Washington State University, Richland, Washington, USA; 3 Department of Entomology, Ecotoxicology Program, Washington State University, Puyallup, Washington, USA

**Keywords:** acetylcholinesterase, carbamates, conservation, organophosphates, pesticides, risk assessment, salmon, synergy, toxicity

## Abstract

**Background:**

Mixtures of organophosphate and carbamate pesticides are commonly detected in freshwater habitats that support threatened and endangered species of Pacific salmon (*Oncorhynchus* sp.). These pesticides inhibit the activity of acetylcholinesterase (AChE) and thus have potential to interfere with behaviors that may be essential for salmon survival. Although the effects of individual anticholin-esterase insecticides on aquatic species have been studied for decades, the neurotoxicity of mixtures is still poorly understood.

**Objectives:**

We assessed whether chemicals in a mixture act in isolation (resulting in additive AChE inhibition) or whether components interact to produce either antagonistic or synergistic toxicity.

**Methods:**

We measured brain AChE inhibition in juvenile coho salmon (*Oncorhynchus kisutch*) exposed to sublethal concentrations of the organophosphates diazinon, malathion, and chlorpyrifos, as well as the carbamates carbaryl and carbofuran. Concentrations of individual chemicals were normalized to their respective median effective concentrations (EC_50_) and collectively fit to a nonlinear regression. We used this curve to determine whether toxicologic responses to binary mixtures were additive, antagonistic, or synergistic.

**Results:**

We observed addition and synergism, with a greater degree of synergism at higher exposure concentrations. Several combinations of organophosphates were lethal at concentrations that were sublethal in single-chemical trials.

**Conclusion:**

Single-chemical risk assessments are likely to underestimate the impacts of these insecticides on salmon in river systems where mixtures occur. Moreover, mixtures of pesticides that have been commonly reported in salmon habitats may pose a more important challenge for species recovery than previously anticipated.

Pesticides are chemical substances that are used to kill, repel, or regulate the growth of biological organisms. This diverse group includes insecticides, herbicides, fungicides, nematicides, acaricides, rodenticides, avicides, wood preservatives, and antifoulants. The U.S. Environmental Protection Agency (EPA) recently estimated that > 1.2 billion pounds of pesticides are applied to crops, forests, residential areas, public lands, and aquatic areas in the United States each year (Kiely et al. 2004). The release of these chemicals into the environment creates a potential for unintended adverse health impacts to both humans and nontarget wildlife.

Mixtures of pesticides are common in the human food supply [National Research Council (NRC) 1993]. Pesticide mixtures are also common in the aquatic environment, including lakes, river, streams, and other surface waters that support aquatic life (Gilliom 2007). Assessing the cumulative toxicity of pesticides in mixtures has therefore been an enduring challenge for environmental health research (Monosson 2005) as well as ecotoxicology (Eggen et al. 2004) for the past several decades. In 1996 the U.S. Congress passed the Food Quality Protection Act (FQPA 1996), which directs the U.S. EPA to assess the human health risks from cumulative exposures to pesticides that share a common mechanism of action. Consideration of mixture toxicity is also required when pesticide tolerances are reassessed under the Federal Food, Drug, and Cosmetic Act (1938). At present, for aquatic species there are no equivalent mandates for consideration of mixture toxicity under the Federal Insecticide Fungicide and Rodenticide Act (1972) or in the development of aquatic life criteria under the Clean Water Act of 1972 (Clean Water Act 1972; Lydy et al. 2004).

The cumulative toxicologic impacts of pesticide mixtures is of particular concern for salmon and steelhead populations that are currently listed as either threatened or endangered under the U.S. Endangered Species Act (ESA 1973). Many wild salmon stocks are in decline across much of the western United States [Nehlsen et al. 1991; NOAA (National Oceanic and Atmospheric Administration) Fisheries 2008]. Past salmon population extinctions (Nehlsen et al. 1991) and current declines have been caused by decades of habitat degradation, overharvest, hydro-power operation, and hatchery practices (NRC 1996). Major river systems that drain large agricultural and urban areas in California, Oregon, Washington, and Idaho provide freshwater habitat for ESA-listed salmon and steelhead (Figure 1). Extensive surface water monitoring for pesticides, as part of the U.S. Geological Survey (USGS) National Water Quality Assessment program (NAWQA), has shown that current-use pesticides are frequently detected in these salmon-supporting river systems (Table 1) (see also more recent monitoring studies by Carpenter et al. 2008; USGS 2008; Washington State Department of Ecology 2008). Furthermore, pesticides almost always occur in mixtures with other pesticides. Analysis of NAWQA monitoring data found that > 90% of water samples from urban, agricultural, and mixed-use streams contained two or more pesticides (Gilliom 2007). The toxicologic effects of these mixtures on the health of salmon are largely unknown.

In the years since the enactment of the FQPA, the U.S. EPA has identified several classes of pesticides that share a common mode of action (U.S. EPA 2002). Among these are the organophosphate (OP) and *N*-methyl carbamate (CB) insecticides. These two classes of chemicals inhibit the enzyme acetylcholinesterase (AChE), thereby interfering with cholinergic neurotransmission in both humans (Chambers 1992) and fish (Fulton and Key 2001). Because anticholin-esterase agents share a common mode of toxic action, the National Academy of Sciences recommended a dose-additive approach to assessing risks to human infants and children (NRC 1993). Dose addition (or, for waterborne exposures to fish, concentration addition) assumes that the cumulative toxicity of the mixture can be estimated from the sum of the individual toxic potencies of each individual component chemical. This is how the U.S. EPA currently assesses the potential toxicity of mixtures of OP and CB insecticides in the context of the FQPA.

The assumption of dose addition or concentration addition for mixtures of anti-cholinesterase pesticides has also been extended to aquatic life (Junghans et al. 2006). In salmon, concentration-additive inhibition of brain AChE activity by mixtures of OP and CB insecticides was recently demonstrated *in vitro* (Scholz et al. 2006). However, the *in vivo* toxicity of anti cholinesterase mixtures may deviate from concentration addition if the individual chemicals in a mixture interact via toxicokinetic or toxicodynamic processes to produce either antagonistic or synergistic effects (Borgert et al. 2004). Each of these possible outcomes (antagonism, addition, or synergism) has potentially important implications for the current regulatory paradigm, wherein risks of pesticides to ESA-listed salmonids are assessed based primarily on responses to single active ingredients. To define the extent to which OP and CB insecticides in mixtures interact, we exposed juvenile coho salmon (*Oncorhynchus kisutch*) to all possible binary combinations of the OP insecticides diazinon, malathion, and chlorpyrifos and the CB insecticides carbaryl and carbofuran. We used the concentration–response curves for AChE inhibition by individual chemicals to statistically define concentration addition (i.e., no interaction within a mixture).

## Materials and Methods

### Fish

Coho salmon eggs were obtained from the University of Washington hatchery (Seattle, WA) and raised at the Northwest Fisheries Science Center’s hatchery (NWFSC, Seattle, WA). Juveniles were maintained at the Washington State University Puyallup Research and Extension Center (Puyallup, WA) for the duration of the study. Fish were held in recirculating tanks of dechlorinated municipal water (hatchery water; temperature 11–13°C, pH 7.0–7.5, dissolved oxygen 90–100%, total hardness as CaCO_3_ 110–120 mg/L, and alkalinity 74 mg/L) on a 12-hr light–dark schedule. Fish were fed commercial salmon pellets (Bio-Oregon, Warrenton, OR) daily. Fish used in experiments were 4–7 months of age with an average size ± SD of 4.9 ± 1.0 cm and 1.3 ± 0.9 g. Experiments followed guidelines set by Washington State University’s Institutional Animal Care and Use Committee for the humane treatment of fish to alleviate suffering during exposures and dissections.

### Pesticide exposures

Diazinon (CAS No. 333-41-5; 98% pure), malathion (CAS No. 121-75-5; 98% pure), chlorpyrifos (CAS No. 2921-88-2; 98% pure), carbaryl (CAS No. 63-25-2; 99% pure), and carbofuran (CAS No. 1563-66-2; 99% pure) were purchased from Chem Service (West Chester, PA). Exposure concentrations used for both single-pesticide and mixture exposures are shown in Table 2. Pesticide-containing stock solutions were prepared in methanol (or ethanol for chlorpyrifos) and added in 100-μL aliquots to 25 L hatchery water in 30-L glass aquaria. Final carrier concentration in exposure tanks was ≤ 0.0004% of the total volume. For each treatment, eight individual fish were exposed for 96 hr on a 24-hr static renewal schedule. Animals were not fed during the exposure interval. After exposures, fish were terminally anesthetized by immersion in MS-222 (tricaine methanesulfonate, 5 g/L; Sigma Chemical Co., St. Louis, MO) until gill activity ceased. Brain tissues were removed, put into plastic microcentrifuge tubes, and kept on ice until stored in a cryogenic (–80°C) freezer for subsequent analyses of AChE enzymatic activity. In the three mixture exposures where mortality was observed, dead fish were removed after 24 hr of exposure and processed for AChE analysis as described above.

### Analytical chemistry

Water samples were collected in 500-mL amber glass bottles from exposure tanks immediately after pesticide addition. All analyses were conducted at Washington State University’s Food and Environmental Quality Laboratory, following existing U.S. EPA methods. Measured pesticide concentrations were generally between 80 and 120% of nominal concentrations (Table 3). For single-chemical exposures, water samples collected at 96 hr indicated only a modest loss (average 5–14%) in pesticide concentration over the course of a 24-hr renewal interval. Accordingly, for subsequent exposures to mixtures, water samples were collected only at *t* = 0. Throughout this article, pesticide exposures are reported in terms of nominal concentrations. Levels of chlorpyrifos in single-pesticide exposures were not determined analytically, because a recent study (Sandahl et al. 2005) characterized the concentration–response relationship between nominal (and measured) chlorpyrifos exposure and brain AChE inhibition in juvenile coho across a range of concentrations identical to the nominal chlorpyrifos exposures used here (0–2.5 μg/L). Thus, in the present study, we used AChE activity relative to published results (Sandahl et al. 2005) to confirm accurate dosing.

### AChE enzyme assays

Determination of AChE activity followed the Ellman method (Ellman et al. 1960) as modified by Sandahl and Jenkins (2002). Briefly, whole brains were homogenized at 50 mg/mL in 0.1 M sodium phosphate buffer (pH 8.0) with 0.1% Triton X-100. Homogenates were centrifuged, and 15 μL of the supernatant was combined with 685 μL of 10 mM phosphate-buffered saline, 50 μL of 6 mM DTNB [5,5′-dithio-bis(2-nitrobenzioc acid)], and 30 μL of 75 mM acetylthiocholine iodide. All chemicals were from Sigma. Triplicate 200 μL samples were transferred to a 96-well plate, and the change in absorbance (at 412 nm) was measured at 12-sec intervals for 5 min at 25°C on an Optimax plate reader (Molecular Devices, Sunnyvale, CA). AChE activity was quantified as milli optical density (mOD) per minute per gram of tissue and reported as a percentage of the baseline enzyme activity for fish exposed to carrier alone.

### Statistics and data analysis

Statistical analyses were performed with either Prism 4.0 (GraphPad, San Diego, CA) or KaleidaGraph (Synergy Software, Reading, PA) software. Tests included nonlinear regression to fit curves of AChE activities, one-way analysis of variance (ANOVA) followed by Dunnett’s post hoc test to establish differences between groups, and one-sample *t*-test with a Bonferroni correction to test for differences between means and predicted values. To allow for a Gaussian distribution of the error around the estimate of median effective concentration (EC_50_), the nonlinear regression performed by Prism 4.0 uses log transformations of the concentrations and reports an estimate of the log transformation of EC_50_.

### Defining toxicologic inter actions between pesticides

**Figure 2:**
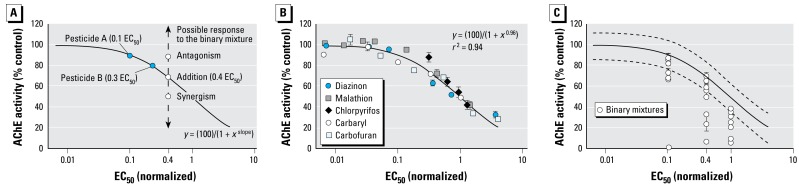
Binary pesticide mixtures cause additive or synergistic AChE inhibition. (*A*) Hypothetical plot describing the three possible toxicologic responses after exposure to a binary mixture of anticholinesterase pesticides. The curve represents a single regression fit to the EC_50_-normalized data from single pesticide exposures. (*B*) Plot of the concentration–response data from five single pesticide exposures after normalization to their respective EC_50_ concentrations and collectively fitting with a nonlinear regression. This curve was used to evaluate the toxicologic response of subsequent binary mixtures (*C*). Values are mean ± 1 SE (*n* = 8). (*C*) Plot of the brain AChE activities of fish exposed to the five pesticides in all possible binary combinations. Based on a default assumption of concentration addition, the pairings were predicted to yield AChE inhibitions of 10% (0.1 EC_50_), 29% (0.4 EC_50_), and 50% (1.0 EC_50_). Values are mean and SE (*n* = 8); dashed lines indicate the 95% prediction band (where 95% of the data should fall based on the regression).

We used significant departures from additive toxicity to define antagonistic and synergistic interactions between pesticides in mixtures (Hertzberg and MacDonell 2002). Addition, in turn, describes an outcome of no interaction, where the predicted toxicity of the mixture (as measured by brain AChE inhibition) is the sum of each chemical’s predicted toxicity (toxic potential). We determined the individual toxic potential for all five pesticides empirically from the concentration–response relationship for AChE inhibition in single-chemical trials. The concentration of each pesticide was normalized to the respective EC_50_ concentration (the concentration estimated to produce a 50% decrease in AChE activity relative to carrier controls) for that individual chemical. The EC_50_-normalized data for all five pesticides were subsequently combined and fit with a single regression. A hypothetical example of a resulting curve and its application to assessing mixture interaction is shown in Figure 2A. For a mixture containing two pesticides at 0.1 and 0.3 EC_50_ units, respectively, concentration addition occurs if the cumulative AChE inhibition is equivalent to 0.4 EC_50_ units. This outcome would fall on the curve or within the 95% prediction band for the regression. Results falling significantly above the curve (less than expected inhibition) would be antagonistic, and results falling significantly below the curve (more than expected inhibition) would be synergistic. In this way, the curve fit to the data from single-chemical trials was used as a basis for detecting interactions between OP and CB pesticides in mixtures.

## Results

### Single pesticides

Exposure to individual pesticides for 96 hr resulted in sublethal, concentration-dependent decreases in brain AChE activity among juvenile coho salmon. No mortality was observed at any of the single-chemical exposure concentrations. We found no significant differences in baseline AChE activity between unexposed fish and those exposed to carrier alone (five one-way ANOVAs, *p* > 0.26). Therefore, AChE activity in pesticide-exposed fish is expressed as a percentage of carrier controls. The mean AChE activity (± SD) for all controls (*n* = 109 animals) was 121.49 ± 13.18 mOD/min, or 14.7 ±1.6 μmoL/min/g. Concentration–response relationships were fit using a nonlinear regression. The equation and resulting curve fit parameters are reported in Table 4. Despite the potential for variability from toxico kinetic differences in absorption, distribution, metabolism, and excretion, the slopes of the concentration–response curves were not significantly different (average = 0.96, *F* test, *p* = 0.1). The relative potencies of the five chemicals did vary, with chlorpyrifos > carbofuran > malathion > diazinon > carbaryl (Table 2; EC_50_ values for AChE inhibition). The EC_50_-normalized data for all five pesticides were then fit with a single nonlinear regression (*r*^2^ = 0.94; Figure 2B). As noted above, this curve was used as a basis to quantitatively determine whether specific binary combinations of pesticides produce interactive (i.e., antagonistic or synergistic) toxicity.

### Pesticide mixtures

Based on a default assumption of dose addition, the five pesticides were combined in all possible pairings to yield predicted AChE inhibitions of 10%, 29%, and 50% in the brains of exposed coho salmon. As determined by the regression in Figure 2B, these levels of enzyme inhibition would result from exposure to 0.1, 0.4, and 1.0 EC_50_ units, respectively. All binary pesticide combinations produced toxicity that was either additive or synergistic, with the frequency of synergism increasing at higher exposure concentrations (Figure 2C). In all cases, the joint toxicity from the paired exposures resulted in AChE activities that were significantly lower than carrier controls (one-way ANOVA; *p*< 0.05).

**Figure 3:**
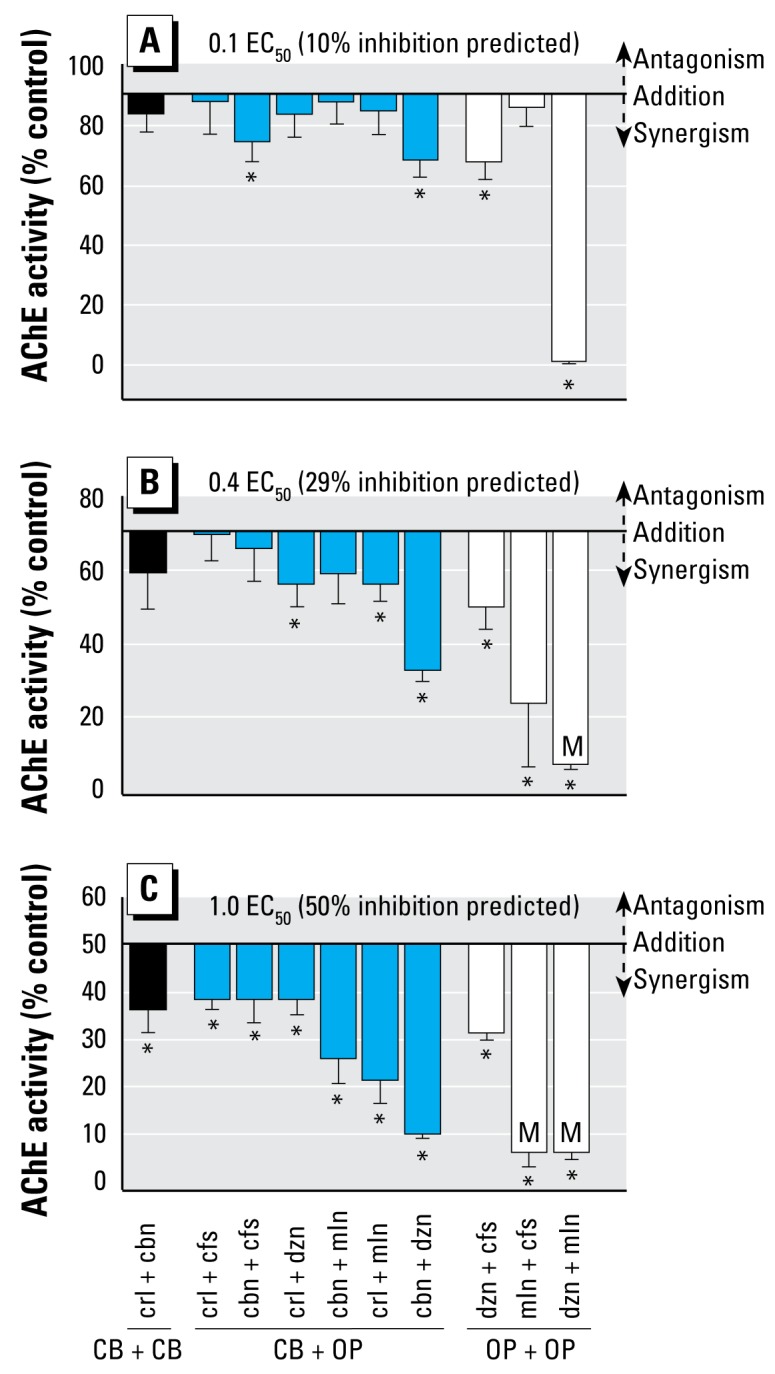
Degree of AChE inhibition in the response to binary combinations of OP and CB pesticides. (*A* ) 10% (0.1 EC_50_). (*B*) 29% (0.4 EC_50_). (*C*) 50% (1.0 EC_50_). OP–OP pairings tended to be more synergistic than other pairings, producing 100% mortality (M) at concentrations that were sublethal in single pesticide exposures. The number of combinations that were statistically synergistic (*t*-test with Bonferroni correction, denoted by asterisks) increased with increasing exposure concentrations. Bars are means (*n* = 8), and error bars indicate the 95% CIs of the mean.

The degree of AChE inhibition in response to specific combinations of OP and CB pesticides is shown in Figure 3. For each of the three EC_50_ units (0.1, 0.4, and 1.0), the measured values for AChE activity are plotted as bars that originate from a horizontal line indicating a (noninteractive) dose-additive response. All combinations of insecticides at each of the three sets of concentrations deflected downward, indicating a tendency toward synergism. Most pesticide pairs yielded rates of enzyme activity significantly lower than would be expected based on concentration addition (*t*-test with Bonferroni correction, *p* < 0.005). The number of statistically synergistic combinations (indicated by asterisks in Figure 3; 20 of 30 pairings overall) increased with increasing exposure concentrations. Additionally, pairings of two OPs produced a greater degree of synergism than mixtures containing one or two CB insecticides. This was particularly true for mixtures containing malathion together with either diazinon or chlorpyrifos. At the highest exposure concentration (1.0 EC_50_), the toxicity of every insecticide mixture was synergistic.

Coho exposed to combinations of diazinon and malathion (1.0 and 0.4 EC_50_), as well as chlorpyrifos and malathion (1.0 EC_50_), had the lowest measured AChE activities. Many fish species die after high rates of acute brain AChE inhibition (> 70–90%; Fulton and Key 2001). As expected from these previous studies, 100% mortality was observed within the first 24 hr among coho exposed to the above pesticide combinations. Fish exposed to these OP mixtures also showed qualitative signs of anti-cholinesterase toxicity, including loss of equilibrium, rapid gilling, altered startle response, and increased mucus production. Although we observed no mortality among coho exposed to the lowest combinations of diazinon and malathion (0.1 EC_50_), all of the fish in this treatment group displayed overt symptoms of sublethal cholinergic poisoning by the end of the 96-hr exposure interval. Therefore, biochemical indicators of synergism (greater than additive AChE inhibition) were consistent with classical signs of anticholinesterase intoxication and death for salmon exposed to mixtures of OP pesticides.

## Discussion

We have shown that *in vivo* exposures to binary mixtures of OP and CB pesticides produced additive or synergistic AChE inhibition in the brains of juvenile coho salmon. The statistical departure from dose addition occurred for several chemical combinations at each of the three relative exposure concentrations, with a trend toward a higher incidence of synergism at the higher exposures. Where the degree of synergism was severe (e.g., for pairings of diazi-non and malathion), enhanced AChE inhibition (i.e., > 90%) corresponded to overt signs of anticholinesterase intoxication and death. This result is consistent with previous (single-chemical) OP and CB pesticide toxicity studies in other fish species (reviewed by Fulton and Key 2001). At present, diazinon, chlorpyrifos, malathion, carbaryl, and carbofuran are some of the most extensively used insecticides in California and the Pacific Northwest (California Department of Pesticide Regulation 2008). The frequency with which these chemicals are detected in some salmon habitats (Table 1) and their combinatorial toxicity to juvenile salmon when they occur as mixtures suggest they may be limiting the recovery of several threatened and endangered populations.

The OP (oxon metabolites) and CB insecticides examined in this study do not interact *in vitro*, where their combinatorial inhibition of salmon AChE can be explained by simple concentration addition (Scholz et al. 2006). The departure from concentration addition for some pesticide pairings *in vivo* is consistent with OP and CB insecticides acting on other biochemical targets. Although more work is needed to identify these targets, car-boxylesterases (CaEs) are candidate enzymes that may underlie the chemical interactions observed in this study. CaEs play an important role in the detoxification of many pesticides, including the OP and CB insecticides, via hydrolysis (Jokanovic 2001; Wheelock et al. 2005a). CaEs may also functionally protect AChE from insecticide toxicity by direct binding and sequestration, thereby preventing or delaying interaction between the insecticide and AChE (Jokanovic 2001; Maxwell 1992a). Mammalian studies spanning several decades have shown that anticholinesterase toxicity increases when CaE enzyme activity is inhibited (Casida et al. 1963; Jokanovic 2001; Maxwell 1992b; Su et al. 1971). Although few studies are documented in fish, exposures to OP and CB pesticides have been found to reduce liver CaE activity in salmonids (Ferrari et al. 2007; Wheelock et al. 2005b), with the OP chlorpyrifos acting as a more potent inhibitor of CaE activity than AChE activity (Wheelock et al. 2005b). In another aquatic species (*Daphnia magna*), pharmacologic inhibition of CaE significantly enhanced the toxicity of chlorpyrifos, malathion, and carbofuran (Barata et al. 2004). Thus, although other biochemical targets may be involved in OP and CB synergism (Casida and Quistad 2004), future mechanistic studies should give particular consideration to the role of CaEs in the pesticide interactions observed in this study.

To identify interactions between pesticides in mixtures, it was first necessary to normalize each concentration–response curve using the calculated EC_50_ concentration for that individual chemical. For all five insecticides, the concentrations that produce 50% brain AChE inhibition in salmon (Table 2) are approximately 10- to 1,000-fold higher than the levels typically detected in surface water monitoring investigations (Hoffman et al. 2000). However, we show here that many insecticide combinations produce additive toxicity at low, environmentally relevant concentrations (0.1 EC_50_; Figure 3A). Moreover, certain combinations showed a clear pattern of synergism even at these relatively low levels. For example, diazinon and chlorpyrifos were synergistic when combined at 7.3 μg/L and 0.1 μg/L, respectively. Surface water monitoring in the San Joaquin basin in California (Dubrovsky et al. 1998) reported diazinon concentrations as high as 6.0 μg/L and chlorpyrifos levels up to 0.5 μg/L. The pairing of diazinon (7.3 μg/L) with malathion (3.7 μg/L) produced severe (> 90%) AChE inhibition as well as classical signs of anticholinesterase poisoning. Thus, for some chemical combinations, synergism is likely to occur at exposure concentrations below the lowest levels used in the present study. Although more work is needed to determine the lower bounds for pesticide interactions, this study indicates that synergism is likely to occur at concentrations that have been directly measured in habitats supporting threatened and endangered salmonids.

In quantitative terms, we have shown that an *in vivo* screen for interactions between anticholinesterase insecticides is tractable in juvenile salmon. Although we examined only five pesticides, it would be straightforward to establish concentration–response relationships for AChE inhibition for the remaining OP and CB insecticides in current use. Given default assumptions of common mode of action and concentration addition (Lambert and Lipscomb 2007), the relative potency of each insecticide could then be used to estimate the joint toxicity of chemicals in a mixture using a conventional toxic unit approach (Junghans et al. 2006). Widely used insecticides and those with a relatively high toxic potency (e.g., the OP azinphos-methyl) could also be screened for interactions with other insecticides at low, environmentally realistic exposure concentrations. Where synergism occurs, additional safety factors could then be assigned to protect the health of threatened and endangered salmon. With the exception of safety factors for synergism, this process is similar to how the FQPA mandates evaluating the human health risks of OP and CB mixtures (FQPA 1996).

Although habitat degradation is generally accepted to be a major causal factor in salmon declines (NRC 1996), the specific contributions of current-use pesticides to the decline of salmon populations are not well understood. One key challenge to understanding this relationship is linking pesticide effects on individual fish to the intrinsic productivity of populations. Recent data by Sandahl et al. (2005) began to address this challenge by showing that exposures to low, environmentally realistic concentrations of chlorpyrifos produced reductions in AChE activity that were closely correlated to reductions in swimming speed and feeding rates. Reductions in feeding are likely to lead to reductions in the size of exposed salmon at the time of their seaward migration, an end point that has been shown to be an important determinant of individual salmon survival (Higgs et al. 1995; Zabel and Achord 2004). By reducing survival rates, sublethal inhibition of AChE in juvenile salmon could potentially reduce the intrinsic productivity of salmon populations. Because mixtures of OP and CB insecticides produce dose-additive or synergistic AChE inhibition, they could magnify these population-scale effects.

The link to populations is important because most of the ongoing recovery planning for ESA-listed salmon is focused at this biological scale (Ruckelshaus et al. 2002). Although many salmon habitats are affected by agrochemicals and urban runoff, restoration priorities are usually developed without the specific inclusion of toxics in quantitative analyses of limiting factors (Bartz et al. 2006; Burnett et al. 2007; Hoekstra et al. 2007; Scheuerell et al. 2006). In the larger context of salmon conservation, a future priority will be to establish a quantitative connection between the mixture toxicity observed in this study and higher biological scales via effects on growth and survival. This connection will help to bridge the disciplines of ecotoxicology and conservation biology (Hansen and Johnson 1999) in their common goal of guiding the recovery of threatened and endangered species.

## Conclusion

These results have important implications for ecological risk assessments, particularly those that focus on the toxicity of individual chemicals as the basis for estimating impacts to imperiled aquatic species. Although the importance of multiple stressors is widely recognized in aquatic ecotoxicology (Eggen et al. 2004), pesticide mixtures continue to pose major challenges for natural resource agencies (Gilliom 2007; Lydy et al. 2004). These challenges include the data gaps that exist for many individual chemicals, experimental design difficulties (e.g., near-insurmountable factorial complexity for large numbers of chemicals), poorly understood pathways for chemical interaction, potential differences in response among species, and the need for more sophisticated statistical tools for analyzing complex data. Salmon exposed to mixtures containing some of the most intensively used insecticides in the western United States showed either concentration-additive or synergistic neurotoxicity as well as unpredicted mortality. This implies that single-chemical assessments will systematically underestimate actual risks to ESA-listed species in salmon-supporting watersheds where mixtures of OP and CB pesticides occur.

## Figures and Tables

**Figure 1:**
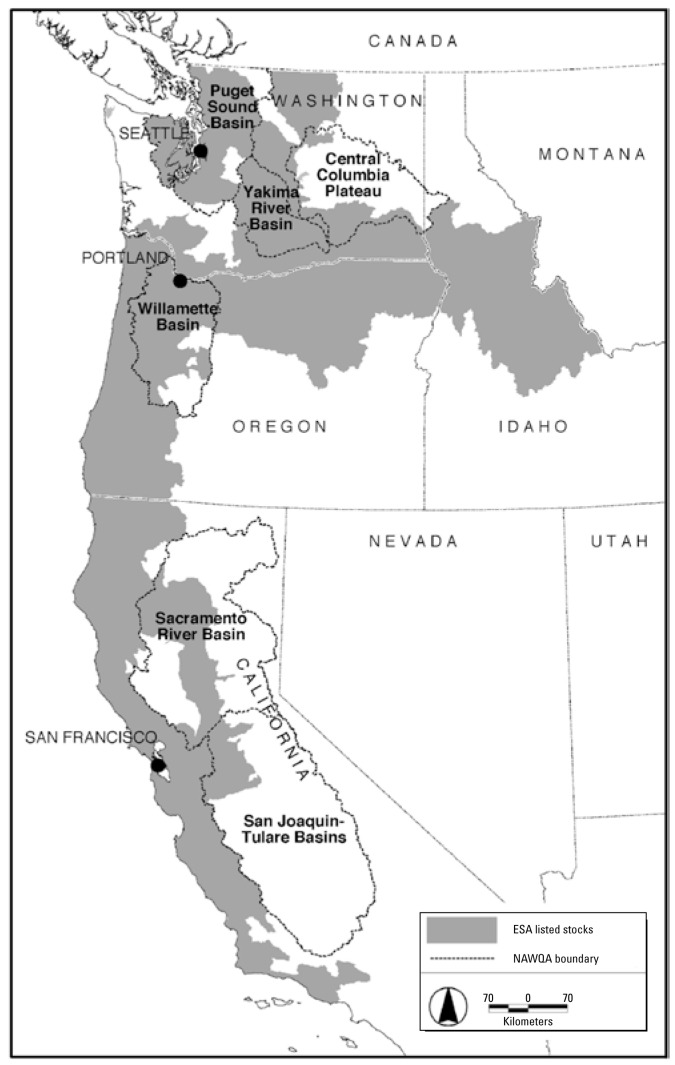
The geographic distribution of threatened and endangered salmon in the western United States overlaps with study units from the USGS NAWQA program. Dashed lines mark the boundaries of NAWQA study areas where pesticide concentrations have been measured in surface waters. Gray shaded areas show the freshwater range of ESA-listed salmon populations.

**Table 1:** Frequency of insecticide detections (% of samples) by the USGS in surface waters of NAWQA basins in the western United States.

NAWQA basin	Diazinon	Malathion	Chlorpyrifos	Carbaryl	Carbofuran
Puget Sound (Ebbert et al. 2000)	48	D	3	D	D
Columbia Plateau (Williamson et al. 1998)	4	2	9	6	5
Yakima River (Fuhrer et al. 2004)	18	D	D	90	ND
Willamette (Wentz et al. 1998)	35	5	21	18	29
Sacramento River (Domagalski et al. 2000)	75	33	38	60	36
San Joaquin (Dubrovsky et al. 1998)	71	8	52	25	5

Abbreviations: D, detected but frequency not reported; ND, not detected.

**Table 2:** Nominal concentrations (μg/L) used in both single-insecticide and mixture exposures.

Insecticide	Single exposures (concentration range)	Mixture exposures			
		1.0 EC_50_	0.5 EC_50_	0.2 EC_50_	0.05 EC_50_
Diazinon	1.0–500	145.0	72.5	29.0	7.3
Malathion	0.5–100	74.5	37.3	14.9	3.7
Chlorpyrifos	0.6–2.5	2.0	1.0	0.4	0.1
Carbaryl	1.0–150	145.8	72.9	29.2	7.3
Carbofuran	1.0–225	58.4	29.2	11.7	2.9

Median effective concentration (EC_50_) values were calculated using nonlinear regressions. For individual chemicals, salmon were exposed to 4–7 concentrations within the indicated range.

**Table 3:** Chemical analysis of insecticide concentrations from both single-insecticide and mixture exposures.

		Mixtures		
Insecticide	Single	1.0 EC_50_	0.4 EC_50_	0.1 EC_50_
Diazinon	88 ± 18	63 ± 5	97 ± 11	106 ± 28
Malathion	89 ± 26	44 ± 27	91 ± 56	70 ± 27
Chlorpyrifos	NA	72 ± 7	79 ± 17	121 ± 15
Carbaryl	113 ± 21	118 ± 33	112 ± 9	108 ± 9
Carbofuran	115 ± 32	112 ± 12	130 ± 15	105 ± 6

Abbreviations: EC_50_, median effective concentration; NA, not analyzed. Values are average percent recovery (relative to nominal concentrations) ± 1 SD.

**Table 4:** Parameters of the concentration–response curves after *in vivo* exposure to individual insecticides.

Insecticide	Log EC_50_ (± SE)^a^	*R*^2^	Slope ± SE
Diazinon	2.2 ± 0.1	0.95	−0.79 ± 0.15
Malathion	1.90 ± 0.05	0.97	−1.32 ± 0.20
Chlorpyrifos	0.30 ± 0.02	0.98	−1.50 ± 0.17
Carbaryl	2.2 ± 0.1	0.95	−0.81 ± 0.16
Carbofuran	1.80 ± 0.09	0.95	−0.82 ± 0.15

a SE of the nonlinear regression; EC_50_ values are presented as μg/L.
